# Efectividad de una solución irrigante en el manejo de absceso periapical crónico: reporte de un caso

**DOI:** 10.21142/2523-2754-1104-2023-180

**Published:** 2023-12-28

**Authors:** Cristian Camilo Morales-Lastre, Julaisy María Cabarique-Mojica, Diana Luz Escobar-Ospino, Jorge Homero Wilches-Visbal

**Affiliations:** 1 Programa de Odontologia, Facultad de Ciencias de la Salud, Universidad del Magdalena. Santa Marta, Colombia. cristianmoralescl@unimagdalena.edu.co, julaisycabariquemm@unimagdalena.edu.co, descobar@unimagdalena.edu.co, jwilches@unimagdalena.edu.co Universidad del Magdalena Programa de Odontologia Facultad de Ciencias de la Salud Universidad del Magdalena. Santa Marta Colombia cristianmoralescl@unimagdalena.edu.co julaisycabariquemm@unimagdalena.edu.co descobar@unimagdalena.edu.co jwilches@unimagdalena.edu.co

**Keywords:** pulpa dental, lesión apical, absceso periapical, irrigantes del conducto radicular, hipoclorito de sodio, dental pulp, apical injury, periapical abscess, root canal irrigators, sodium hypochlorite

## Abstract

El medio endodóntico posee condiciones óptimas para el crecimiento de microorganismos que pueden liberar subproductos hacia la región periapical del diente y ocasionar lesiones inflamatorias. La desinfección química mediante el uso de irrigantes desempeña un papel clínico importante, toda vez que estos son capaces de eliminar desechos de tejidos orgánicos e inorgánicos, lo que genera un efecto antibacteriano residual. El propósito de este trabajo fue mostrar la efectividad del hipoclorito de sodio al 2,5% y el 5,25% como agente irrigante para el manejo clínico de un absceso periapical crónico en un diente con reabsorción apical. Inicialmente, se utilizó hipoclorito de sodio al 2,5%; sin embargo, al no tener una respuesta óptima de cicatrización del absceso después de 3 días, se optó por utilizar una concentración al 5,25% para lograr un mejor efecto bactericida. Después de 5 días, se logró la cicatrización de la fistula y se continuó con el tratamiento endodóntico. Una de las perspectivas de este trabajo es investigar más sobre el empleo de antibioticoterapia en conjunto con un buen protocolo de irrigación.

## Abstract (en)

The endodontic environment has optimal conditions for the growth of microorganisms that can release by-products into the periapical region of the tooth and cause inflammatory lesions. Chemical disinfection using irritants solutions plays an clinical important role, as they are able to remove waste from contaminated organic and inorganic tissues, generating a residual antibacterial effect. The purpose of this study was to show the effectiveness of 2.5% and 5.25% sodium hypochlorite as an irrigating agent for the clinical management of a chronic periapical abscess in a tooth with apical resorption. Initially, 2.5% sodium hypochlorite was used, however, as it did not have an optimal abscess healing response after 3 days, it was decided to use a concentration of 5.25% to achieve a better bactericidal effect. After 5 days, the fistula healed, and endodontic treatment continued. One of the perspectives of this case report is to investigate more about the use of antibiotic therapy in conjunction with a good irrigation protocol.

## INTRODUCCIÓN

El medio endodóntico posee condiciones óptimas para el crecimiento de microorganismos anaerobios y gramnegativos, que pueden afectar el tejido pulpar ^1^^,^^2^. Si esto no se trata adecuadamente, los microorganismos pueden liberar subproductos que se desplazan hacia la región periapical del diente y ocasionan lesiones periapicales inflamatorias ^3^. La presencia de radiolucidez periapical es el signo clínico principal de estas lesiones, las cuales pueden desarrollar distintas condiciones patológicas, como abscesos periapicales, granuloma periapical y quiste radicular ^4^^,^^5^.

Los abscesos periapicales son respuestas inflamatorias que adoptan los tejidos perirradiculares por causa de una infección de origen pulpar ^6^. Se distinguen en agudos y crónicos. Si el agudo persiste, se vuelve crónico después de cierto tiempo ^6^^,^^7^. Los abscesos crónicos se caracterizan por la extravasación de material purulento a través de fistulas ligadas a un tracto sinuoso intraoral o extraoral, y suelen ser asintomáticos ^6^^,^^7^.

La eliminación de los microorganismos es el principal objetivo del tratamiento endodóntico. Existen determinadas variantes anatómicas que presentan algunos sistemas de conductos radiculares (conductos accesorios y delta apicales), las cuales dificultan su desinfección mecánica ^8^^-^^10^. Por tanto, la desinfección química a través del uso de irrigantes desempeña un papel importante. Estos son capaces de eliminar desechos de tejidos orgánicos e inorgánicos, y de lubricar las paredes de la dentina, lo que genera un efecto antibacteriano residual ^9^. Entre los distintos tipos de irrigantes se encuentran el hipoclorito de sodio, la clorhexidina, los quelantes, la solución salina, etc. ^8^ ^9^.

El hipoclorito de sodio se considera el irrigante de primera elección, debido a su acción antimicrobiana por tener un pH alcalino, capacidad de disolver residuos de tejidos orgánicos y acción lubricante ^8^^,^^9^. Podemos encontrarlo en distintas concentraciones que van desde el 0,5% al 8,25%; sin embargo, todavía no hay consenso sobre la concentración en la cual se evidencie su mayor efectividad ^9^. Según estudios recientes ^9^^,^^11^, la efectividad varía según su concentración. Las concentraciones más altas pueden proporcionar ventajas; no obstante, pueden amplificar efectos indeseables de la solución, entre ellos la disminución del módulo de elasticidad, la resistencia a la tracción o a la flexión, y la microdureza de la dentina ^9^. 

El propósito de este trabajo fue demostrar la efectividad del hipoclorito de sodio como agente irrigante en concentraciones del 2,5% y el 5,25%, para el manejo clínico de un absceso periapical crónico en un órgano dentario posteroinferior con reabsorción apical. Según la literatura, las concentraciones del 2,5% son tan efectivas como las del 5,25%.

## REPORTE DEL CASO

**Figura 1:**
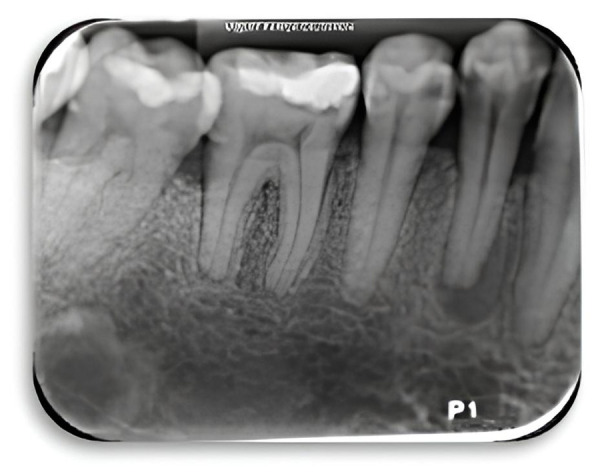
Imagen radiográfica preoperatoria de los órganos dentales 44, 45 y 46.

Paciente femenino de 36 años acude al servicio odontológico de la Universidad del Magdalena, refiriendo molestia en el primer premolar inferior derecho y sin antecedentes médicos relevantes. A la inspección clínica, se observó gingivitis asociada con biopelícula dental en un periodonto reducido, y presencia de edema intraoral fluctuante sin tracto fistuloso a nivel del órgano dental 44. Asimismo, se evidencia una lesión en forma de cuña de tipo abfracción en tercio cervical y desgaste en la cúspide vestibular. Se establece diagnóstico presuntivo, trauma oclusal en órgano dental 44. Posteriormente, se realizaron pruebas de sensibilidad pulpar obteniendo como resultado: negativo al frío, positivo a percusión vertical y negativo a percusión horizontal. Radiográficamente, se evidenció lesión y reabsorción radicular de 2 mm aproximadamente en región periapical (Fig. 1). 

**Figura 2:**
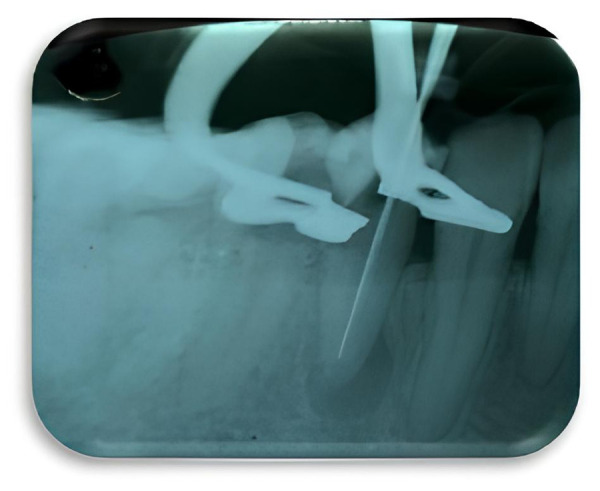
Imagen radiográfica de instrumentación del órgano dental 44.

**Figura 3:**
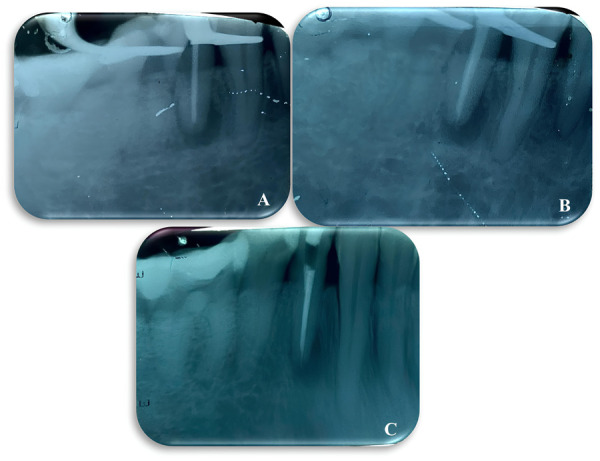
A) Imagen radiográfica de conometría. B) Imagen radiográfica de control de obturación. C) Imagen radiográfica de corte final.

**Figura 4:**
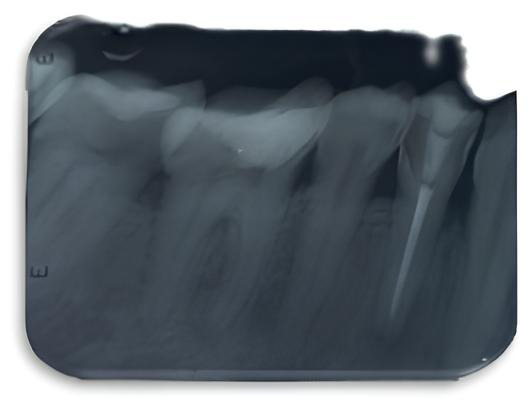
Imagen radiográfica final

El diagnóstico definitivo del órgano dental 44 fue absceso periapical localizado. Desde entonces, se inició su tratamiento endodóntico que consistió en lo siguiente: 

1. Técnica anestésica infiltrativa y del nervio lingual, utilizando 1½ de lidocaína al 2% con epinefrina 1:80.000.

2. Aislamiento absoluto de la pieza dentaria usando dique de hule (nic tone, España), grapa 00 (Hu-Friedy, EE. UU.) y arco de Young (Hu-Friedy, EE. UU.). 

3. Conformación de cavidad de acceso ovoidal con fresa redonda Nº 2 (Diatech, EE. UU.) y fresa Endo Z (Maillefer, EE. UU.) para forma de conveniencia. 

4. Preparación biomecánica del conducto radicular utilizando la técnica crown down, mediante instrumentación manual utilizando limas tipo K (Maillefer, EE. UU.) (Fig. 2). Entre cada lima se realizó limpieza y desinfección del conducto siguiendo un protocolo de irrigación con hipoclorito de sodio al 2,5%, almacenado en una jeringa (Monoject, España) de 5 ml con aguja de salida lateral calibre 27G, a una longitud tentativa de trabajo (LTT) de 18,5 mm. La irrigación fue activada realizando movimientos de impulsión y tracción dentro del conducto por cinco minutos con una lima 15 (Maillefer, EE. UU.).

5. Debido a la lesión apical presente, se realizó medicación intracanal con hidróxido de calcio por 3 días y se selló la cavidad temporalmente con oxifosfato de zinc (Lee Smith, Colombia).

6. Después de 3 días de medicación, se evidenció la presencia de tracto fistuloso en la mucosa circundante al órgano dental. Se retiró el cemento temporal y se aumentó la concentración del hipoclorito de sodio al 5,25%, con el fin de incrementar sus efectos bacteriostáticos y bactericidas. Mediante un protocolo de activación (punto 4), el hipoclorito actuó por 10 minutos dentro del conducto, a medida que se realizaban movimientos de entrada y salida con una lima 15 tipo k (Maillefer, EE. UU.) cada 2 minutos. 

7. Posteriormente, se realizó un curetaje del tracto fistuloso con cureta endodóntica para drenar. Se colocó una gasa estéril embebida con clorhexidina (Periogard, Colombia) en la mucosa circundante y se dejó medicación intracanal con hidróxido de calcio (Eufar, Colombia) por 3 días.

8. Luego de 3 días, se observó aún la presencia de fístula en la mucosa circundante; por tanto, se efectuó nuevamente el protocolo de irrigación mencionado anteriormente (punto 4). No obstante, después de 5 días se evidencio la cicatrización del tracto fistuloso.

9. Posteriormente, se realizó la obturación del órgano dentario mediante la técnica de condensación lateral e, inmediatamente, la cavidad fue sellada temporalmente con cemento de óxido de zinc y eugenol (Proquident, Colombia) (Figs. 3A, 3B y 3C).

10. Finalmente, en los 3 días siguientes se llevó a cabo la restauración del órgano dentario con resina compuesta A2 Filtek z250 (3M, Colombia) mediante técnica incremental y fotopolimerizándola por 20 segundos. Igualmente, se realizó la restauración de la lesión en forma de cuña a nivel de tercio cervical. Por último, se dio acabado y pulido final a la restauración (Fig. 4).

**Figura 5:**
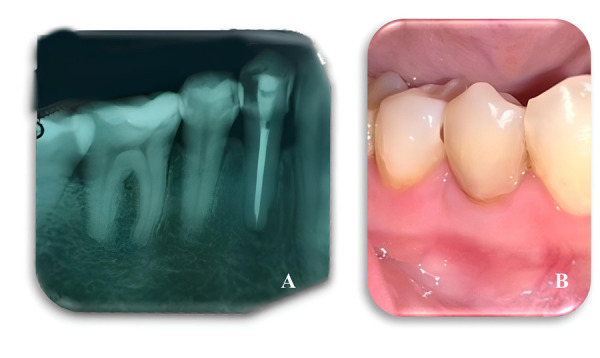
A) Radiografía de control después 10 meses. B) Tejidos blandos circundantes.

Después de 10 meses, se realizó un seguimiento clínico al órgano dentario, en el cual se evidencia un óptimo proceso de cicatrización a nivel de tejidos periapicales (Fig. 5A). Por otra parte, a nivel de tejidos blandos circundantes, se observa buena cicatrización, sin presencia de fístula (Fig. 5B).

### Declaración de aspectos éticos

Los autores declaran que los procedimientos cumplen los principios éticos de la Declaración de Helsinki ^12^ y la Resolución 8430 de 1993 ^13^ del Ministerio de Salud de Colombia. El paciente autorizó por medio del consentimiento informado la utilización de fotos e información para fines clínicos o investigativos. La información entregada fue custodiada acorde a la Ley Estatutaria 1581 de 2012.

## DISCUSIÓN

Los abscesos periapicales se originan debido a una infección en el área del conducto radicular y representan una reacción del organismo para protegerse frente a la invasión de microorganismos ^(7, 14)^. Un elemento significativo en el desarrollo de esta enfermedad es la alteración en el equilibrio de las bacterias orales, que se desplaza hacia la disbiosis de la población microbiana ^15^. La finalidad de la terapia endodóntica es realizar la limpieza y desinfección del sistema de conducto radicular, para abarcar todos los tejidos de la pulpa, tanto vital como necrótica, así como los microorganismos y subproductos microbianos afectados. Dado que el sistema de conducto radicular es altamente complejo y variable, su limpieza y desinfección de manera predecible se ven limitadas ^16^. 

El uso exclusivo de diversas técnicas de instrumentación resulta ineficaz para lograr conductos radiculares libres de bacterias. Por consiguiente, se vuelve imperativo emplear un irrigante endodóntico en conjunto con la instrumentación mecánica para contribuir a la desinfección y lubricación del conducto radicular, eliminando los residuos del sistema de conductos y disolviendo los tejidos orgánicos e inorgánicos ^17^. La irrigación durante el abordaje clínico de infecciones de origen endodóntico es considerada como el factor de éxito del tratamiento ^18^. El hipoclorito de sodio es considerado el estándar de oro para irrigantes endodónticos debido a su potente actividad antimicrobiana y capacidad para disolver tejidos orgánicos. Estas propiedades han respaldado su uso en endodoncia desde la década de 1920 ^17^.

Se han utilizado concentraciones de hipoclorito de sodio para la irrigación del conducto radicular, variando del 0,5% al 6,0%. Al respecto, no existe convergencia sobre su actividad antimicrobiana: algunos defienden la prescripción de concentraciones elevadas (a partir del 10%), pese a su toxicidad; mientras que otros recomiendan concentraciones bajas ^19^. 

Por otra parte, el estudio de Cué-Díaz *et al*. ^20^ evaluó el uso y la efectividad del hipoclorito de sodio al 1% en el tratamiento endodóntico del absceso periapical crónico, y se obtuvo resultados similares al estudio mencionado anteriormente. Se formaron dos grupos distintos: en el grupo experimental, conformado por 19 pacientes, se utilizó hipoclorito de sodio al 1%, y en el grupo control, compuesto por 20 pacientes, se usó suero fisiológico al 0,9%. Se llevaron a cabo evaluaciones clínicas de los pacientes de ambos grupos a las 72 horas y a los 7 días, cuando se repitió el análisis bacteriológico y se aplicaron los mismos irrigantes. En más del 50% de los pacientes evaluados, el hipoclorito de sodio al 1% demostró una efectividad moderada, puesto que se observó una mejora clínica razonable al reducir significativamente los síntomas y la carga de microorganismos endodónticos en solo una semana. 

Se sugiere que, a mayor concentración de hipoclorito de sodio, se logrará una mayor disolución tisular y una mejor neutralización de la toxicidad en el conducto. Sin embargo, es importante tener en cuenta que las concentraciones más altas pueden afectar y causar irritación en los tejidos apicales y periapicales. Por esta razón, se sugiere utilizar una concentración del 5,25% y trabajar con precaución para no dañar dichos tejidos ^21^.

Los estudios anteriores demostraron la eliminación de microorganismos en el mismo intervalo de tiempo mediante el hipoclorito de sodio a distintas concentraciones (1% y 2%). En el presente trabajo, inicialmente, se utilizó hipoclorito de sodio al 2,5%; sin embargo, al no tener una respuesta óptima de cicatrización del absceso, después de 3 días (aparición de fistula) se optó por utilizar una concentración al 5,25%, a fin de lograr un mejor efecto bactericida. Después de 5 días se logró la cicatrización de la fístula y se continuó con el tratamiento endodóntico hasta su posterior restauración estética. 

## CONCLUSIONES

El uso del hipoclorito al 2,5% es empleado comúnmente en los protocolos de irrigación cuando se presentan casos de lesiones apicales. En este caso, al no obtener una evolución positiva por parte de la lesión, se aumentó la concentración del irrigante al 5,25%, la cual permitió la resolución del cuadro clínico en 5 días. En este sentido, se recomienda la adición de antibioticoterapia en conjunto con un buen protocolo de irrigación para el manejo clínico de los abscesos periapicales crónicos. 

## Financiamiento:

Ninguno
